# Volatile Constituents, Inorganic Elements and Primary Screening of Bioactivity of Black Coral Cigarette Holders

**DOI:** 10.3390/md9050863

**Published:** 2011-05-18

**Authors:** Xueting Bai, Yicun Chen, Weizhou Chen, Huaping Lei, Ganggang Shi

**Affiliations:** 1 Department of Pharmacology, Shantou University Medical College, Shantou 515041, China; E-Mails: bxt19840819@163.com (X.B.); chenyicun@yeah.net (Y.C.); 2 Marine Biology Institute, Shantou University, Shantou 515063, China; E-Mail: wzchen@stu.edu.cn; 3 School of Life Sciences, Sun Yat-sen University, Guangzhou 510275, China; E-Mail: audenlei@163.com

**Keywords:** black coral cigarette holders, CO_2_-SFE, antioxidant activity, hydroxyl radicals, antimicrobial activity

## Abstract

Black corals (BC) have been used for a long time in Chinese medicine, and may have some pharmaceutical functions when used as material for cigarette holders in southeast China. This study is aimed to investigate the bioactivities of volatile constituents in BC and to explore the folklore behind the use of BC cigarette holders (BCCHs). We extracted the volatile constituents of BC by supercritical fluid extraction (SFE) with carbon dioxide (CO_2_-SFE), then identified and analyzed the constituents by gas chromatography-mass spectrometry (GC-MS). In total, 15 components were reliably identified in BC and found to be biologically active. These included triethyl phosphate, butylated hydroxytoluene, cedrol, *n*-hexadecanoic acid, squalene, and cholesterol. Meanwhile 13 inorganic elements (P, Ca, Mg, S, B, Si, Fe, Cu, Zn, Ba, *etc.*) were determined by inductively coupled plasma spectrometer (ICPS). In the bioactivity tests, the BC extract (BCE) showed a scavenging activity of 2,2-diphenyl-1-picrylhydrazyl free radicals and hydroxyl radicals by phenanthroline-Fe (II) oxidation and moderate inhibition of Gram-positive microorganisms. The antioxidant and antimicrobial activities of BC, which are related to the active chemical composition, may explain the perceived benefit for cigarette smokers who use BCCHs.

## 1. Introduction

Black coral (BC) belongs to the *Antipathes*, which, interestingly means “against suffering”, and are a group of rare, tree-like corals found in deep water. BC has been harvested for centuries for both ornamental and medicinal purposes, by people of many cultures who believe that BC has the power to ward off evil and injury. In China, *Myriopathes japonica* (Brook, 1889) [1], one genera of BC, is commonly called Hailiu and is regarded as “sacred trees”. It is used to prevent bleeding, relieve pain and stop convulsions [2]. Traditionally, *Myriopathes japonica* was widely used as an ornament on belts or pendants. However, it has been optimized for use as cigarette holders, owing to its natural features, the structure of skeletal ring growths and inability to rot or damage [3].

Cigarette holders have a long history. Many modern cigarette holders come with a cigarette filter system, which can be helpful in filtering coke tar and nicotine. Various materials for cigarette holders are available such as wood, jade, bakelite, ivory and precious metals (gold, silver and platinum). Black coral cigarette holders (BCCHs), made of marine natural biomaterials, are one characteristic product of the Chaozhou-Shantou area in Guangdong province of China, and have been widely used by Chaoshan people for a long time. Anecdotally, besides absorbing detrimental substances existing in tobacco like other cigarette holders, BCCHs could give off a special fragrance that helps eliminate bad breath and clear sore throats. According to the descriptions of some smokers with bronchitis, using BCCHs can help resolve phlegm, relieve coughing and moisturize the lungs.

When smokers inhale, a cigarette burns at 700 °C at the tip and around 60 °C in the core. This heat breaks down the tobacco to produce various toxins in distinct groups for later solidification and a gas phase. More than 4000 chemical compounds are contained in cigarettes, including at least 400 toxic substances [4]. The best known solid substances are nicotine and tar. Equally toxic ones, if not more so, are the chemicals, particularly oxidants in the gas phase, such as radicals, hydrogen peroxide, peroxynitrate, and peroxynitrite [5]. Considerable research has documented the oxidative damage of biological micromolecules caused by tar radicals, oxidants, and free radicals in cigarette smoke [6]. Also, tobacco use can also increase susceptibility to bacterial infections, such as respiratory infections, periodontitis and bacterial meningitis [7–9]. Moreover, evidence indicates that bacterial infection, as a cause of exacerbations, may be attributed to the pathogenesis of chronic obstructive pulmonary disease (COPD) [10].

At present, there are only three literature references about the chemical constituents extracted from black corals. Aiello *et al.* [11] reported five new highly oxygenated sterols isolated from the black coral *Antipathes subpinnata* through brine shrimp assay. Qi *et al.* [12,13] found a new carbazole alkaloid showing weak cytotoxicity toward human liver carcinoma, from the South China Sea black coral *Antipathes dichotoma*. Thus far, little is known about the volatile constituents extracted by supercritical fluid methods, inorganic elements of BC, and the antioxidant and antimicrobial properties of CO_2_-SFE (supercritical fluid extraction) extracted materials of black coral (EBC) as well.

Hence, we wondered whether BCCHs have antioxidant and antimicrobial bioactivities that could reduce the effects incurred by cigarette smoking, because a much higher temperature during inhalation may also release some volatile constituents from BCCHs. Some active chemicals contained in BCCHs may be associated with the properties of BCCH. Therefore, in this study, we investigated the volatile constituents of BCCHs extracted by supercritical fluid with carbon dioxide (CO_2_-SFE) and identified by gas chromatography-mass spectrometry (GC-MS). Moreover, *in vitro* antioxidant and antimicrobial activity was studied to evaluate the biological activities of BC extract (BCE).

## 2. Results and Discussion

### 2.1. Supercritical Fluid Extraction and Gas Chromatography-Mass Spectrometry (GC-MS)

**Figure 1.**
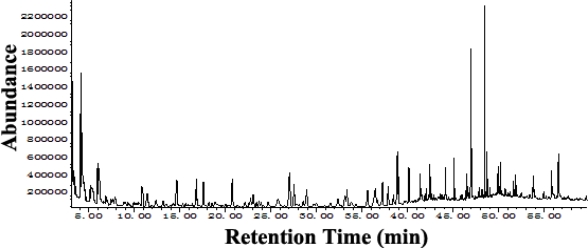
The total ion chromatogram of the volatile constituents from black coral by supercritical fluid with carbon dioxide (CO_2_-SFE).

For investigation of the volatile compounds of BCCHs, we used CO_2_-SFE for extraction and separation. The analysis of essential oils is usually conducted with GC-MS to characterize and identify volatile organic compounds in complex samples [14]. GC-MS combined with retention index (RI) can provide more reliable qualitative analysis of complicated essential oil samples than GC-MS alone because the chromatographic peaks are separated satisfactorily [15]. So compared by mass spectra, the temperature-programmed RIs of these compounds were calculated based on the formula. The RI values were very similar to the RIs reported in the literature, with a margin of a few units (6–7 at most). The total ion chromatogram (TIC) of the volatile constituents from the CO_2_-SFE is shown in Figure 1. The abundance of each peak was not high in the middle of the TIC because of the low concentration of splitless injections. In total, 15 constituents identified are shown in Table 1, including alkanes, terpenes, esters, fatty acids, and aromatic compounds. Phenol, 2,2′-methylenebis[6-(1,1-dimethylethyl)-4-methyl- was the most abundant compound.

The extraction of volatile components using conventional processes, such as solvent extraction, is conducted at high pressure and temperature and is time-consuming. SFE is a rapid, selective, effective and convenient technique for sample preparation before the analysis of compounds. Supercritical fluids have been used as solvents for a wide variety of applications such as essential oil extraction, and more than 90% of all analytical SFE is performed with CO_2_ [16]. With low pressure and temperature and no influence of solvent, use of CO_2_-SFE is a good way to extract the volatile constituents of BCCH for the process of smoking. Thus, because of the volatilized constituents it is not difficult to understand why the color of BCCH can be changed after being used for a period of time.

Of the 15 components identified by GC-MS, some possess biological activities, such as triethyl phosphate, which has anticholinesterase activity [17]. The antioxidant activity of squalene is known, and the compound is now used for vaccine delivery applications and for enhancing the immune response [18]. As a widely used antioxidant food additive, butylated hydroxytoluene (BHT) has potential toxicity. Several reports indicated neoplasm-promoting activity, but Williams *et al.* concluded that there was no cancer hazard of BHT at the current food additive levels, and it may actually be anticarcinogenic at levels as low as 100 ppm [19]. Some hydrophobic compounds have been reported to have antimicrobial activity, such as thujopsene [20]. Furthermore, cedrol can induce autonomic responses in humans, such as decreasing the heart rate and blood pressure [21]. The characteristic odors and special fragrance of BCCH are due not only to the BC coming from the deep sea but also the compounds contained in them, such as hydrocarbons and oxygenated compounds [22]. The compounds related to the odor of BCCH may sooth a smoker’s throat. Thus, investigations here preliminarily investigated the bioactive substances existing in BC, which can contribute to the functional properties of BCCH. Additional work is necessary to fractionate and identify the constituents of the extracts further to elicit a better understanding of unknown constituents.

### 2.2. Element Analysis

The elemental analysis of BCCH yielded 13 components as shown in Table 2. Concentrations of P and Ca were higher than other elements in the samples reported here. Ba was present in the lowest concentration. As we know, phosphorus (P) is an essential element for the formation of skeleton; black corals are no exception. More than 60% of skeletal P is incorporated as intracrystalline organic phases. The phosphorus to calcium ratio of coral skeleton approximates that of seawater [23].

The essential role of trace elements is clear in biological systems, especially the metal elements. They are immensely important for redox balance and antioxidant function. Trace elements such as Cu, Fe, Mn, Se, and Zn act as cofactors of antioxidant enzymes to protect the body from oxygen free radicals produced during oxidative stress. For instance, the redox trace-copper is essential in the regulation of the antioxidant enzymes Cu/Zn-superoxide dismutase [24]. Some sulfur-containing antioxidant compounds, such as cysteine, methionine, taurine, glutathione, lipoic acid, mercaptopropionylglycine, *etc.* play key roles in oxygen free radical formation [25]. Metal chelators such as ceruloplasmin play an important function to contain the reactive Cu ion. Alcoholic compounds such as carveol may remain complexed with transition metals like Mn, Zn, *etc.* [26], improving antioxidant status using trace element supplementation [27]. Consequently, the bioactivities of trace elements in the function of BCCH should be researched further.

### 2.3. Antioxidant Property Assays of Extract

**Figure 2.**
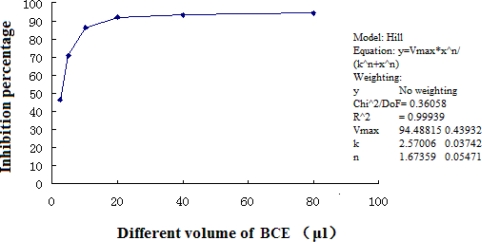
The black coral extract (BCE) showed dose-dependent DPPH^•^ scavenging activity.

Table 3 shows the DPPH scavenging activity of BCE. As a positive control, vitamin C (VC) was also examined. Assessed samples were able to reduce the stable violet DPPH^•^ to the yellow DPPH-H, with 50% inhibitory concentration (IC_50_) values of 2.57 μL (2248.75 μg/μL). The BCE showed dose-dependent DPPH^•^ scavenging activity (Figure 2).

Reactive oxygen species (ROS), including free radicals, oxygen ions, and peroxides, are implicated in cell damage. The use of DPPH^•^ provides an easy, rapid and convenient way to evaluate the antiradical activities of antioxidants independent of sample polarity [28]. The interaction of a potential antioxidant with DPPH^•^ depends on its structural conformation. Certain compounds react rapidly with DPPH^•^ corresponding to the number of available hydroxyl groups, like the positive control VC. However, the mechanism of most compounds is more complex [19]. Some compounds need a longer reaction time. Thus, during the testing of DPPH radical-scavenging activity, the absorbance values were measured every half hour. The percentage inhibition of DPPH^•^ was increased with all BCE doses, except at the highest dose, up to 1.5 h later, when inhibition showed no further change.

**Figure 3.**
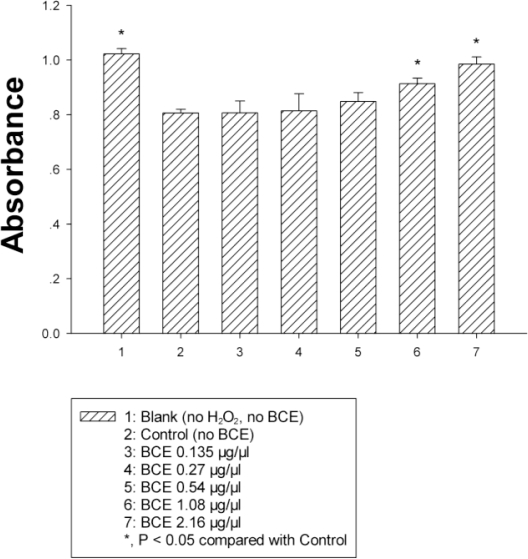
The absorbance increase of hydroxyl radical-scavenging corresponding to the concentration of the black coral extract (BCE).

**Figure 4.**
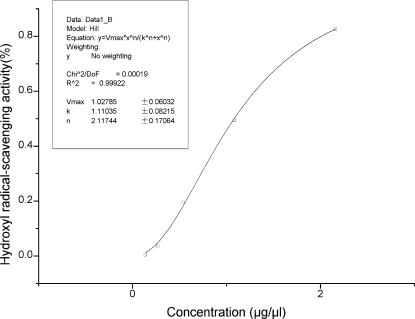
The hydroxyl radical-scavenging activity (%) of black coral extract (BCE).

As one of the ROS produced in biological systems, HO^•^ can cause DNA, protein, and lipid oxidation [29]. The transition metal catalyzing Fenton reaction is one of the most widely accepted mechanisms for HO^•^ production, in which ([Fe(o-phen)_3_]^2+^) can be oxidized by hydrogen peroxide to generate HO^•^. Consequently, the absorbance of 1,10-phenanthroline-iron (II) complex ([Fe(o-phen)_3_]^2+^) was tested here at 536 nm. After adding H_2_O_2_, ([Fe(o-phen)_3_]^2+^) was oxidized by H_2_O_2_ to generate HO^•^. The absorbance of the control sample (no BCE) was the lowest due to the decreasing ([Fe(o-phen)_3_]^2+^). The addition of antioxidants can prevent ([Fe(o-phen)_3_]^2+^) from oxidation by hydrogen peroxide, so that the efficiency of the scavenging hydroxyl radical can be determined. Compared to the controls, an increased absorbance was observed of which the magnitude corresponded to the concentration of BCE (Figure 3). The antioxidant compounds in BCE could reduce the oxidation of HO^•^, and the absorbance of BCE groups was close to the blank by chelating reaction of Fe^2+^ and phenanthroline. The BCE was capable of inhibiting the hydroxyl radical greatly in a concentration-dependent fashion. As can be seen in Figure 4, with increasing concentration of BCE, the inhibitory effect was enhanced greatly. The hydroxyl radical-scavenging activity (%) of max concentration was 82.7%.

The oxidants and prooxidants contained in cigarette smoke can produce ROS and enhance oxidative stress, which can further cause lipid peroxidation [30]. Several articles have reported that smokers should use some antioxidants to prevent the formation of ROS and oxidative damage [31,32]. Specifically, oral administration of natural antioxidant, such as vitamin C [33–35], vitamin E [36,37], and extractions of tea [38,39], fruits [40] and marine algae [41] may reduce this damage. Our results demonstrate that the components contained in BC have scavenging abilities of DPPH^•^ and HO^•^. The antioxidative properties of BCE may be attributed to the presence of the active compounds we identified, such as BHT, cedrol, squalene and fatty acids, *etc.* It has long been known that phenolic compounds are effective antioxidant agents that act as free radical terminators [42]. Phenolic compounds have the ability to quench free radicals because of both their acidity (alcoholic hydroxyl group) and their delocalized π-electrons of the benzene ring [43]. In terms of the compound phenol, 2,2′-methylenebis[6-(1,1-dimethylethyl)-4-methyl-, the higher percentage of relative amount to total may be related to antioxidant activity of BCE. BHT, known as a lipid-soluble antioxidant, is closely related to phenol antioxidants. Each BHT consumes two peroxy radicals [44]. Now BHT has been marketed as a health supplement for food and pharmaceuticals. Squalene acts mainly as a peroxyl radical scavenger. The ability of squalene to quench singlet oxygen is reported to be similar to BHT [45]. Squalene was also found to be a much stronger scavenger of hydroxyl radicals than endogenous reduced glutathione [46]. Accumulated evidence demonstrates the antioxidant activity of essential fatty acid components extracted from various plants. Therefore, tetradecanoic acid, *n*-Hexadecanoic acid and oleic acid present in BCE may contribute to the antioxidant activity. Oxidative stress results from an imbalance between excess oxidants and/or a depletion of antioxidants [47,48]. Cigarette smoke, the major risk factor for the development of COPD, contains more than 10^14^ free radicals per puff [49,50]. In addition, the antioxidants of BCCH may be present in the inhaled tobacco smoke. Using BCCH could retain a balance of oxidants and antioxidants and benefit smokers by lessening the damage caused by cigarettes through antioxidant release.

### 2.4. Antimicrobial Susceptibility Testing

The antimicrobial activity of BCE against the tested strains was assessed qualitatively by the presence or absence of inhibition zones. The BCE exhibited moderate antimicrobial activity against the Gram-positive microorganisms (Table 4).

In general, no effect was observed against Gram-negative microorganisms. Gram-positive bacteria are more susceptible to essential oils than Gram-negative bacteria [51]. *S. aureus* and *S. epidermidis* are sensitive to fatty acids [52]. The weak antibacterial activity against Gram-negative bacteria is ascribed to the presence of an outer membrane, which possesses hydrophilic polysaccharide chains as a barrier to hydrophobic essential oils [53]. CO_2_-SFE mainly extracts the lipid fraction, which cannot go through the outer membrane barrier. The results of the minimum inhibitory concentration (MIC) determination indicated the BCE inhibited the microorganisms tested. *S. aureus* and *S. epidermidis* displayed a MIC of 73 and 37 μg/μL, respectively.

The risk of microbial infections can be significantly increased by cigarette smoking including respiratory infections, periodontitis and bacterial meningitis [17]. A number of free fatty acids are known to possess antibacterial activity against Gram-positive bacteria, as well as their esters [54,55]. Some synthetic fatty acid analogs of cholesterol show excellent antibacterial activity *in vitro* [56]. The presence of the hydroxyl group of chemical composition, especially phenolic components, such as thujopsene, can destabilize the bacterial membrane to increase the ability of antimicrobials. The antimicrobial activity and therapeutic efficacy of oleic acid in a liposomal formulation against methicillin-resistant *S. aureus* has been proven [57]. Meanwhile, some compounds with a small percentage, such as palmitic acid, have been reported to have significant antimicrobial activity [58]. The components at low concentrations might be involved in some type of synergism with the other active compounds [59]. *S. aureus* is one common Gram-positive bacterium found in nosocomial pneumonia and a potentially life-threatening infection. However, the incidence of infections is due to *S. epidermidis* [60]. Our BC extract showed antimicrobial activity by inhibiting the growth of both *S. aureus* and *S. epidermidis*.

## 3. Experimental Section

### 3.1. Sample Collection

The study area, Nan Ao Island, is on the Tropic of Cancer, located 116°56′–117°09′E and 23°23′–23°29′N off the city of Shantou, eastern Guangdong Province, neighboring Fujian Province, between Hong Kong and Taiwan. This region is at the northern edge of coral distribution in East Asia [61]. The raw materials used in the experiments were obtained from the manufacturer of Hailiu cigarette holders in Nan Ao Island. BC powder was collected during the processing of BCCHs.

**Figure 5.**
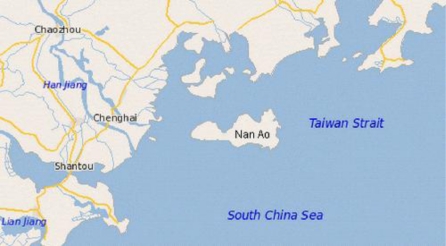
The geographical position of the locality of Nan Ao Island.

The geographical position of the investigated locality is shown in Figure 5.

### 3.2. Chemicals and Reagents

Industrial alcohol (95%), hexane (pesticide grade), methanol (HPLC grade), (analytical grade), *n*-alkane standard solutions of C8–C20 (mixture No. 04070) and C21–C40 (mixture No. 04071) were from Fluka Chemika (Buchs, Switzerland). 2,2-Diphenyl-1-picrylhydrazyl (DPPH) was obtained from Sigma–Aldrich (St. Louis, MO, USA). 1,10-phenanthroline and FeSO_4_ were acquired from Bio Basic Inc. (Canada). All the other agents (ethanol, H_2_O_2_, HNO_3_, *etc.*) were of analytical grade. Ultrapure water was used for the experiments.

### 3.3. Supercritical Fluid Extraction with Carbon Dioxide

CO_2_-SFE involved use of the HA221-50-06 extractor (Huaan Supercritical Equipment Co., Nantong, Jiangsu, China). The instrument was run with CO_2_ for both the extraction and cooling gases. The extraction pressure and extraction temperature were 300 bars and 45 °C, respectively.

BC powder was placed into 5 L extraction thimbles, 985 g and 984 g. The samples were extracted with pure CO_2_, with ethanol used as a modifier. The amount of ethanol, 1 L, was approximately equal to the sample volume. Fractions were collected in both separators into 2 separate containers. The addition of a polar co-solvent (ethanol 95%) led to increased efficiency of extraction.

### 3.4. Gas Chromatography-Mass Spectrometry Analysis

GC-MS analysis involved an Agilent 7890 GC equipped with a quadrupole 5975 mass spectrometer (Agilent Technologies, CA, USA) and a HP-5ms capillary column, 30 m × 0.25 mm in diameter, with 0.25 μm film thickness. The column was maintained at an initial temperature of 100 °C for 2 min, then programmed at 10 °C/min from 100 to 140 °C and then at 1 °C/min from 140 to 280 °C, which was maintained isothermal for 5 min. Helium was used as the carrier gas. Samples were injected splitless. The constant helium carrier gas flow was 0.8 mL/min. The temperature of the GC injector and GC-MS interface were maintained at 280 °C. The mass spectrometer was operated in the electron ionization mode (EI, 70 eV) with full scan in the 50–550 *m/z* range.

Identification of the constituents was based on comparison of the obtained mass spectra with those of reference compounds in the database of the NIST Mass Spectral Search Program (NIST 08 mass spectral database, National Institute of Standards and Technology, Washington, DC, USA). The following quasi-linear equation for temperature-programmed retention indices was used:

(1) $$RI_{x}\;=\;[(t_{x}\;-\;t_{n})/(t_{n+1}\;-\;t_{n})]\;\times \;100$$

where *RI_x_* is the temperature-programmed retention index of interest and *t_n_*, *t*_*n*+1_, *t_x_* the retention time in minutes of the 2 standard *n*-alkanes containing *n* and *n* + 1 carbons and index of interest, respectively [62].

### 3.5. Element Analysis

The measurements of multi-elements were performed with a Shimadzu Model ICP-AES1000IV inductively coupled plasma spectrometer (Tokyo, Japan), determined using a peak scanning program. 99.99% argon was used as carrier gas. The powder of black corals (0.2216 g, 0.2347 g and 0.2478 g, respectively) was incubated at 120 °C for 2 h, and digested with nitric acid for about 1 h, 80 °C, and then the yellow solution was diluted with ultrapure water to 100 mL. Concentrations of tested elements were calculated based on the standard curves, which were prepared by stepwise dilution of the standard solutions (1000 μg/mL).

Instrumental details and operating conditions for ICPS: working frequency, 27.12 MHZ; power, 1.2 KW; flow of carrier gas, 1.0 L/min; integration time, 5 s; view heights, 15 cm.

### 3.6. Antioxidant Property Assays of Extracts

Antioxidant assays of scavenging effects on DPPH.

The DPPH method was used as described previously [63]. In total, 2.5, 5, 10, 20, 40, and 80 μL of BCE (875 μg/μL) was added to an ethanol solution of DPPH (final concentration 0.2 mM/mL). The mixture was shaken vigorously and left to stand at room temperature for 30 min. Then, 200 μL of the resulting solution was transferred to 96-well flat-bottomed plates, and the absorbance was measured at 517 nm using a Thermo Labsystems Multiskan Spectrum microplate reader (Waltham, MA, USA). The radical-scavenging activities of samples, expressed as percentage inhibition of DPPH^•^, was calculated according to the following formula: inhibition percentage (*Ip*) = [(*A*_B_ – *A*_A_)/*A*_B_] × 100, where *A*_B_ and *A*_A_ are the absorbance values of the blank sample and resulting solutions, respectively.

Hydroxyl radical-scavenging by phenanthroline-Fe (II) oxidation.

Hydroxyl radical-scavenging was measured by phenanthroline-Fe (II) oxidation assay. ([Fe(o-phen)_3_]^2+^) can be oxidized by hydrogen peroxide to generate HO^•^ by Fenton’s reaction [64]. The reaction mixture contained 80 μL of sample solution at different concentrations, 50 μL (10 mmo1/L) phenanthroline, 50 μL (10 mmo1/L) FeSO_4_ and 50 μL (0.15%) H_2_O_2_, added to 400 μL phosphate buffer (pH 7.27). After 1 h incubation at 37 °C, the absorbance at 536 nm was recorded.

The scavenging activity of hydroxyl radical was expressed by the following equation: hydroxyl radical-scavenging activity (%) = (*A*_S_ – *A*_1_)/(*A*_0_ – *A*_1_) × 100, where AS is absorbance of the sample; *A*_1_ is absorbance of the control solution containing 1,10-phenanthroline, FeSO_4_ and H_2_O_2_; and *A*_0_ is absorbance of the blank solution containing 1,10-phenanthroline and FeSO_4_.

### 3.7. Antimicrobial Susceptibility Testing and Determination of Minimum Inhibitory Concentration (MIC)

Antimicrobial susceptibility testing for BCE involved the Kirby–Bauer (KB) disk diffusion method against Gram-positive and -negative bacteria. The microorganisms included *Staphylococcus aureus* (American Type Culture Collection (ATCC) No. 25923), *Staphylococcus epidermidis* (ATCC 12228), *Escherichia coli* (ATCC 25922) and *Pseudomonas aeruginosa* (ATCC 27853). Briefly, a suspension of the tested microorganism (0.1 mL of 10^8^ cells/mL) was spread on solid media plates. Filter paper discs (6 mm in diameter) were impregnated with 40, 80, 160 μL of BCE and placed on the inoculated plates, then incubated at 37 °C for 24 h.

A broth microdilution method was used to determine the MIC against the susceptible microorganisms using the KB method according to the National Committee for Clinical Laboratory Standards A serial doubling dilution of BCE from 19–292 μg/μL was prepared in 50 μL Mueller Hinton broth (MHB) in each well of a 96-well microtiter plate. Freshly grown microbial suspensions in MHB were standardized to a cell density of 1.5 × 10^8^ (McFarland No. 0.5) and added to the wells (50 μL). After incubation at 37 °C for 24 h, the MIC was defined as the lowest concentration of BCE at which the microorganism did not demonstrate visible growth.

### 3.8. Statistical Analyses

Assays were carried out in triplicate, and each analysis of all samples was performed in duplicate; results displayed are averages. Absorbance values are expressed as means ± SD. Differences between variables were tested by one-way ANOVA with SPSS 13.0 (SPSS Inc., Chicago, IL, USA). A *P* < 0.05 was considered statistically significant.

## 4. Conclusions

This study presented the first data for some volatile constituents identified through CO_2_-SFE, GC-MS analysis, and inorganic elements within BC. Results from analytical experiments and limited biological assessments indicate that the antioxidant and antimicrobial activities of BCCH might be due to the identified chemical composition. Although more research is necessary to evaluate the unknown fraction of the chemical composition and further biological studies of BCCH are needed, evidence suggests that the bioactivity of BCCH can be potentially effective for smokers to reduce oxidative stress and bacterial infections due to smoke toxin exposure.

## Figures and Tables

**Table 1.** Volatile constituents of black coral identified by gas chromatography-mass spectrometry and retention index.

**No.**	**RT ^a^**	**Constituents**	**RI ^b^**	**% ^c^**
1	4.014	Triethyl phosphate	1121.3	3.97
2	9.293	Thujopsene	1436.8	0.19
3	11.407	Butylated hydroxytoluene	1514	1.90
4	14.604	Cedrol	1605.3	1.21
5	22.571	Tetradecanoic acid	1762.1	0.19
6	28.923	1,2-Benzenedicarboxylic acid bis(2-methylpropyl) ester	1861.5	2.24
7	33.340	Hexadecanoic acid, methyl ester	1927.0	1.65
8	36.452	*n*-Hexadecanoic acid	1973.4	3.15
9	41.396	11-Octadecenoic acid, methyl ester	2097.8	1.16
10	42.903	Oleic Acid	2167.5	0.25
11	45.234	Tricosane	2298.6	0.32
12	46.929	Phenol, 2,2′-methylenebis[6-(1,1-dimethylethyl)-4-methyl-	2420.9	4.91
13	48.483	1,2-Benzenedicarboxylic acid, mono(2-ethylhexyl) ester	2550.9	5.00
14	51.638	Squalene	2830.8	0.54
15	55.800	Cholesterol	3077.6	1.92

a Retention times (minute);

b Retention index were calculated from our analyses with respect to a series of *n*-alkenes;

c percentage of relative amount to total.

**Table 2.** Elemental analysis obtained by inductively coupled plasma spectrometer (ICPS).

**Elements determined**	**Concentration (μg/g)**
P	21401.48 ± 2194.83
Ca	13738.4 ± 476.42
Na	3916.90 ± 574.11
S	3691.77 ± 159.52
Mg	1776.84 ± 193.78
Fe	781.80 ± 344.78
Cu	597.60 ± 32.26
Zn	296.19 ± 37.76
K	268.25 ± 137.38
Al	184.47 ± 32.89
Si	71.96 ± 16.09
B	42.16 ± 20.34
Ba	5.81 ± 2.91

**Table 3.** The *in vitro* antioxidant activities of the positive control (vitamin C (VC)) and CO2-SFE of black coral extract (BCE) by DPPH radical scavenging.

**Sample**	**Mean ± SD**
Blank	1.247 ± 0.004
VC, 0.01 mM (1.7 mg/mL)	0.045 ± 0.003 *
BCE, 2.5 μL (875 μg/μL)	0.668 ± 0.016 *
BCE, 5 μL (875 μg/μL)	0.368 ± 0.018 *
BCE, 10 μL (875 μg/μL)	0.171 ± 0.008 *
BCE, 20 μL (875 μg/μL)	0.102 ± 0.007 *
BCE, 40 μL (875 μg/μL)	0.085 ± 0.004 *
BCE, 80 μL (875 μg/μL)	0.074 ± 0.003 *

* *P* < 0.05, compared with blank control.

**Table 4.** The antimicrobial activity of black coral extract against Gram-positive microorganisms showing the diameters of the inhibition zones (mm).

**Bacterial strains**	**BCE (875 μg/μL)**			
	**20 μL**	**40 μL**	**80 μL**	**160 μL**
*Staphylococcus aureus*	6.9 ± 0.4	10.1 ± 0.6	13.8 ± 0.5	17.9 ± 0.3
*Staphylococcus epidermidis*	7.8 ± 0.3	10.0 ± 0.3	11.9 ± 0.2	14.0 ± 0.3
